# Using remote sensing data within an optimal spatiotemporal model for invasive plant management: the case of *Ailanthus altissima* in the Alta Murgia National Park

**DOI:** 10.1038/s41598-023-41607-2

**Published:** 2023-09-04

**Authors:** Christopher M. Baker, Palma Blonda, Francesca Casella, Fasma Diele, Carmela Marangi, Angela Martiradonna, Francesco Montomoli, Nick Pepper, Cristiano Tamborrino, Cristina Tarantino

**Affiliations:** 1 School of Mathematics and Statistics, The University of Melbourne, Parkville, VIC 3010 Australia; 2 Melbourne Centre for Data Science, The University of Melbourne, Parkville, VIC 3010 Australia; 3 Centre of Excellence for Biosecurity Risk Analysis, The University of Melbourne, Parkville, VIC 3010 Australia; 4 Institute of Atmospheric Pollution Research, National Research Council (CNR), Via Amendola 173, 70126, Bari, Italy; 5 Institute of Sciences of Food Production, National Research Council (CNR), Via Amendola 122/O, 70126, Bari, Italy; 6 Istituto per le Applicazioni del Calcolo M. Picone, National Research Council (CNR), Via Amendola 122/I, 70126, Bari, Italy; 7 Department of Mathematics, University of Bari, via Orabona 4, 70125 Bari, Italy; 8 Department of Aeronautics, Imperial College London, Exhibition Road, London, SW7 2AZ UK; 9 The Alan Turing Institute, The British Library, London, UK; 10 Department of Computer Science, University of Bari, via Orabona 4, 70125 Bari, Italy

**Keywords:** Invasive species, Ecosystem services

## Abstract

We tackle the problem of coupling a spatiotemporal model for simulating the spread and control of an invasive alien species with data coming from image processing and expert knowledge. In this study, we implement a spatially explicit optimal control model based on a reaction–diffusion equation which includes an Holling II type functional response term for modeling the density control rate. The model takes into account the budget constraint related to the control program and searches for the optimal effort allocation for the minimization of the invasive alien species density. Remote sensing and expert knowledge have been assimilated in the model to estimate the initial species distribution and its habitat suitability, empirically extracted by a land cover map of the study area. The approach has been applied to the plant species *Ailanthus altissima* (Mill.) Swingle within the Alta Murgia National Park. This area is one of the Natura 2000 sites under the study of the ongoing *National Biodiversity Future Center (NBFC)* funded by the Italian National Recovery and Resilience Plan (NRRP), and pilot site of the finished H2020 project *ECOPOTENTIAL,* which aimed at the integration of modeling tools and Earth Observations for a sustainable management of protected areas. Both the initial density map and the land cover map have been generated by using very high resolution satellite images and validated by means of ground truth data provided by the EU Life Alta Murgia Project (LIFE12 BIO/IT/000213), a project aimed at the eradication of *A. altissima* in the Alta Murgia National Park.

## Introduction

Invasive alien species (IAS) are allochthonous species whose introduction and spread cause severe ecological damages to habitats and species, and reduce biological diversity considerably. IAS severely disrupt natural ecosystems and ecosystem services with consequent adverse environmental, economic, social and cultural implications worldwide^1^ and they are a large anthropogenic threat to ecosystems and biodiversity, fostered by globalisation^2^. Although economic impacts of many IAS are not available, the combined impacts are large. The environmental and economic damages of IAS in the United States, the United Kingdom, Australia, South Africa, India, and Brazil estimated to be in excess of US $ 314 billion per year^3^. IAS economic damage to agriculture, forestry and fisheries, is estimated to be at least EUR 12 billion per year in Europe alone^4^.

Habitat restoration is hard, labour intensive and costly but necessary to safeguard native species from being displaced by IAS because of competition for resources. Nevertheless, improving measures to control IAS impacts is prioritized in current EU biodiversity policy: to address the problem of IAS, reduce and monitor their environmental repercussions, the European Commission approved a specific regulation (Regulation 1143/2014) supporting interventions aimed at prevention, early detection, rapid eradication and management of spreading invasive species. Development and implementation of model-based decision support systems are a key asset for effective eradication and control programmes for IAS^5^. The Italian National Biodiversity Future Centre^6^ focus its research activities in findings suitable strategies for biodiversity conservation and monitoring in order to reach EU targets. Assessing and monitoring biodiversity and its evolution, to study native species at risk of extinction, and to plan protection and conservation interventions are some of its specific tasks. Mathematical models are effective tools for evaluating the impact of IAS on natural ecosystems of crucial value for human activities such as forests, agricultural soils and the agrifood sector^7–10^. Models allow to build scenarios of conservation planning and management. Specifically, we need to model management strategies that incorporate budget constraints and the spatiotemporal spread dynamics of species^11^. Modelling and field testing in pilot areas, as the Alta Murgia National Park (Southern Italy), a Natura 2000 Site, is useful to identify conservation priorities under present and future scenario.

*Ailanthus altissima* (Mill.) Swingle is an invasive plant species that has significant impacts in Europe and worldwide. It reproduces both by seeds dispersal^12^ and asexually by vegetative sprouts^13,14^. The winged seeds can be dispersed by wind, water and machinery^15^, while its robust root system can generate numerous suckers and progeny plants. *A. altissima* typically occurs in very dense clumps, resulting from even-aged seedling establishment or from clonal expansion through root sprouting, and occasionally it grows as widely spaced or single stems. *A. altissima* grows on a broad range of anthropogenic to natural sites, from stony and sterile soils to rich alluvial bottoms^16^. Due to its vigour^17^, rapid growth, tolerance, adaptability and lack of natural enemies, it spreads spontaneously out-competing other plant species and reducing their growth. *A. altissima* causes serious direct and indirect ecological, economic, functional and aesthetic damages in non-crop areas^18^. Above all, it poses a significant threat to biodiversity^19^ and this threat is likely to increase in the future unless robust action is taken at all levels to control its advancement and spread. The costs of controlling and eliminating it amount to billion of euro per year^20^. Population management is difficult because of the production of numerous basal and radical shoots after cut and for a hardly eradicable root system. The best control strategy is based on a combined use of mechanical and chemical methods^21–23^.

In the last decades *A. altissima* has quickly spread in the Alta Murgia National Park, which is mostly characterized by dry grasslands and pseudo-steppe, with wide open spaces and low vegetation, whose tendency is to be easily invaded. *A. altissima* causes serious direct and indirect damages to ecosystems, replacing and altering communities that have a great conservation value^24^, producing severe ecological environmental and economic effects and causing natural habitat loss and degradation. Many plants and infested areas grow in vulnerable natural habitats, on rocky soils, and at forest edges. There are hundreds infested areas (thousands of *A. altissima* trees) in the park. Its presence is scattered within the park, by roadsides, dry stone walls, inside and around jazzi (antique sheep folders) and ruins, and is also highly present close to wells and pools. Thus, we consider that the infestation covers the whole park area, but the “real” infested area could be a small percentage compared to the total surface. While the species has been planted historically, this no longer occurs and the park regulation forbids its introduction. There is no exact record about the starting date of the infestation but, considering the size of some trees, we assume that it started at least 80–100 years ago because farmers living in the area introduced plants for ornamental purposes or to create shade, not aware of their invasive potential. *A. altissima* can seriously harm the ecological balance of the park threatening the fragile grassland ecosystem and its native biodiversity. Only active on-going management can ensure the conservation of the wild flora and fauna species of the park. Hence, the LIFE+ Programme, the European Commission financial instrument for environment and climate action, funded the Life Alta Murgia Project (LIFE12 BIO/IT/000213 https://www.ispacnr.it/progetto-life-alta-murgia/) having as main objective the eradication of the invasive exotic tree species *A. altissima* from the Alta Murgia National Park using innovative and environmentally friendly techniques.

One of the challenges involved in modelling control strategies for *A. altissima* is the lack of detailed and current data on extent and control efficacy. The most recent map of *A. altissima* presence and distribution in the park is from 2012, and was a deliverable of the Life Alta Murgia project. This map pre-dates the on-going eradication program. Due to these data limitations, we propose the use of a relatively simple mechanistic model, a reaction–diffusion model, validated against the current plant spatial distribution by satellite images detection. We incorporate the effect of an eradication program using a reaction term, which simulates the plant abatement during a control program. Within the approaches proposed in literature for modeling the IAS removal^7,10,11,25,26^, we adopt the spatially explicit model^9^, where the rate of removal is described by an Holling II type response function and the best control strategy is obtained by minimizing the environmental and economic dameges in terms of costs, under the realistic hypotesis of limited resources.

The growth of the species in our model is modulated by an habitat suitability index (HSI) function, which is estimated by using remote sensing data. Habitat suitability models help both in understanding species niche requirements and predicting species potential distribution^27^. These models statistically relate field observations to a set of environmental variables (e.g., climate, topography, and soils) to predict spatial potential suitable distributions indicating the suitability of locations for a target species across a landscape or region. In this work, a ranking procedure is applied to obtain an estimation of the HSI function, based on the presence map of *A. altissima* and the land cover map in the analyzed area. Moreover, the continuous Boyce index^27^ is evaluated to validate the results and assess the ability of the estimated HSI map in predicting the presence of the plant.

The value of our predictive mechanistic model is to provide an automatic tool for an a-priori estimate of the effectiveness of a planned control action under temporal and budget constraints. In this study, the interest was focused on finding the best budget allocation both in space and time for the park area maintenance, which can help determine whether a control policy needs to be improved. We identified the function of the costs and we formulate an optimal control problem with a penalty term.

As result, we developed an automatic tool that can get the plant presence information from the satellite and use that information for predicting the best action of the park manager. Indeed, there is a lot of satellite data that are not being used to its full potential and there are optimisation methods that are powerful, but not being used on-ground. We tried to link the two domains and make their combined solution accessible and useful to managers. Specifically, the model ingests the initial presence density of *A. altissima*, obtained from a data-driven classification approach applied to very high resolution (VHR) satellite images, combined with the park land cover map. The latter is also used for providing a rough estimate of habitat suitability. To calculate the optimal budget allocation from control measures, we implemented a numerical procedure based on composition of implicit and exponential Lawson schemes^28^ in a forward–backward algorithm^29,30^. We developed the web service, COINS (COntrol of INvasive Species), as a decision support tool to be integrated into management practices. Data and workflows are available through a virtual research environment (VRE) and public metadata catalogues. The parameter values used in the model were taken both from literature and expert knowledge and estimated by the Life Alta Murgia Project data. However, uncertainty will be associated with these parameter values, regardless of whether they are selected using expert knowledge or inferred from data. Arbitrary Polynomial Chaos^31^ is used to assess the effect of the parametric uncertainty on the predictions of the model. So, the primary aim of our analysis is to assess qualitatively the effectiveness of control strategies for the species *A. altissima* as well as their sensitivity to changes in allocated budget and surveillance efficiency.

## Plant detection

**Figure 1:**
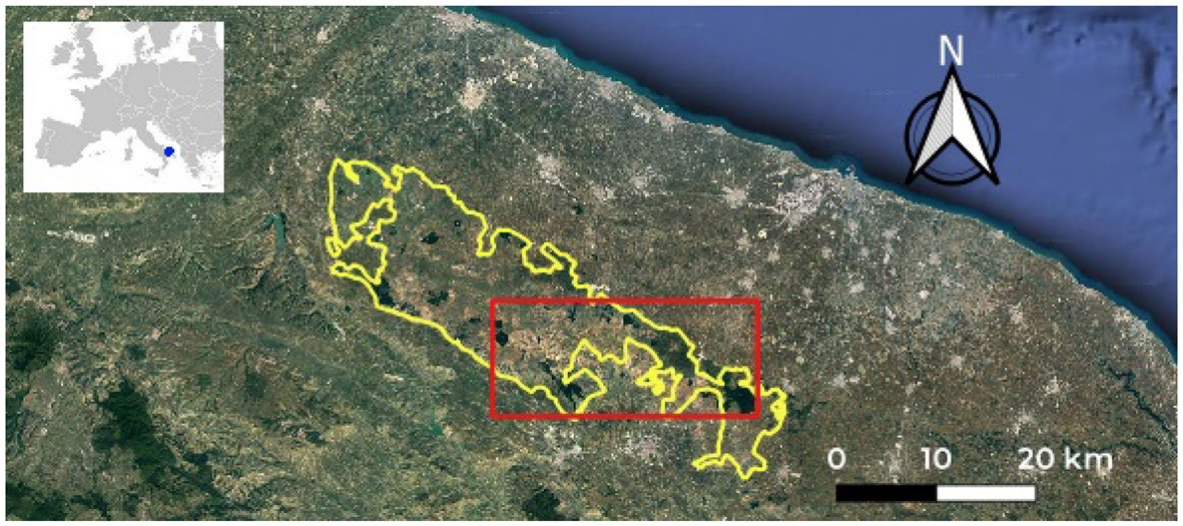
Alta Murgia National Park and study area (500 km$^2$) in yellow and red boundaries, respectively, overlaid on a Google satellite image (https://www.google.it/intl/it/earth/). Map obtained by QGIS 3.24 (https://qgis.org/en/site/).

**Figure 2:**
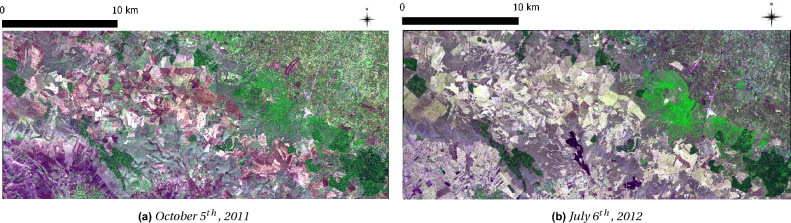
WV-2 input image, 2 m resolution. (**a**) 2011, (**b**) 2012. FalseColour composite: R = 5, G = 7, B = 2. Source: images provided by the European Space Agency data warehouse policy, within the FP7 BIO_SOS project (https://cordis.europa.eu/project/id/263435), GA 263455.

The study area covers 500 km${2}$ partially within the Alta Murgia National Park, which is located in the Natura 2000 network protected area (IT9120007) within the Apulia region (Southern Italy) (Fig. 1). The mapping of *A. altissima *invasive specie was obtained using a set of four, cloud-free, multi-seasonal WorldView-2 (WV-2) satellite imagery, at 2 m of spatial resolution, acquired on May 19th, 2011, October 5th, 2011 (Fig. 2a), January 22nd, 2012 and July 6th, 2012 (Fig. 2b).

The employed technique consisted of a two-stages hybrid classification process: the first applied a knowledge-driven learning scheme to provide a land cover map (LC) including deciduous vegetation and other classes; the second exploited a data-driven classification to discriminate pixels of the invasive species found within the deciduous vegetation layer of the LC map from the first stage. Specifically, the first stage of the classification process was based on an object-oriented, knowledge-driven LC classification algorithm within the eCognition framework (https://docs.ecognition.com/v9.5.0/). The algorithm was developed in a previous study^32–34^. It is based on multi-class discrimination using spectral and context rules provided through the elicitation of prior expert knowledge about agricultural practices, class phenology, spectral and spatial features. In the first stage no training data were needed to produce the output LC map. The input to the first stage were the multi-seasonal WV-2 images available corresponding to: the biomass pre-peak (January), the biomass peak (May), the dry season (July), the biomass post-peak (October). The deciduous vegetation layer, to which *A. altissima* belongs, was extracted from the LC map obtained as output of the first stage and it was used for the masking of the images to be analyzed in the second stage.

The second stage of the algorithm was based on a data-driven, pixel-based support vector machine (SVM) classifier fed in input with the WV-2 images pair acquired in July and October (after testing different input configurations) only for those pixels belonged to the deciduous vegetation layer from the first stage. So the *A. altissima* pixels were distinguished from those belonging to other deciduous vegetation for a two-classes problem and a final binary (*A. altissima*—other deciduous) output mapping for the invasive species was obtained^35^. The accuracy assessment protocol^36–38^ was adopted in the second stage of the system to evaluate the reliability of the *A. altissima* mapping. The overall accuracy (OA) and the user’s accuracy (UA) values for *A. altissima* were 97.96 $\pm$ 0.14% and 82.47 $\pm$ 1.32%, respectively.

An additional processing step was performed by adding a convolution median low-pass filter to the output map. Thus, the levels of detail achieved depended on the filter size used. Although the application of a filtering procedure may remove not only noisy and false positive pixels but also some pixels belonging to isolated *A. altissima* plants, a median filter was adopted to improve the final overall accuracy (OA) value^39^. The choice of the filtering increased OA and UA values up to 99.41 $\pm$ 0.14% and 97.47 $\pm$ 0.01%, respectively, for a $5\times 5$ window size. The Supplementary Information contains the workflow of the hybrid two-stage classification algorithm used to produce the mapping of *A. altissima*.

**Figure 3:**
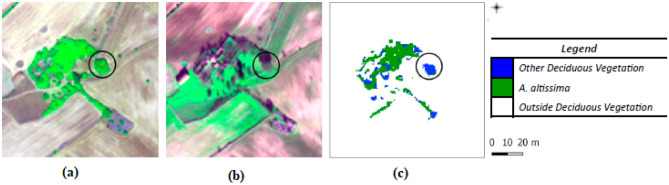
Close-up: (**a**) WV-2 July and (**b**) WV-2 January images, as R = 5, G = 7, B = 2 composite. In the winter image the deciduous vegetation does not appear green. (**c**) *A. altissima* output map from second stage. In the black circle a deciduous shrub correctly distinguished from *A. altissima* by SVM classifier is shown.

The images considered in the present study were provided by the European Space Agency (ESA) under the Data Warehouse 2011–2014 policy within the FP7-SPACE BIO_SOS project (www.biosos.eu). The images were co-registered with each other and calibrated to top of atmosphere (TOA) reflectance values. The reference data used for the training of the second-classification stage and for validation of the final output map were collected during the European Life Alta Murgia Project carried out to eradicate the invasive plant species *A. altissima*^40^. In the Supplementary Information, we show the binary map of the initial presence (2012) of *A. altissima* in the analyzed area.

## Habitat suitability estimation

We used remotely sensed data to generate the habitat suitability index (HSI) map of *A. altissima* in the Alta Murgia National Park, defined as the potentiality of the habitat to host the plant. The map is composed of cells whose values range from 0 to a maximum value of 0.262638. These values indicate how close the local environment is to the species optimal conditions, higher values standing for the most suitable areas. HSI values give a measure of how the growth rate and the carrying capacity vary across the analyzed area^41^. Habitat suitability models use empirical relationships between the species distribution and the features of the environment to give an estimate of the HSI for each area of the landscape. Although remotely sensed variables are traditionally used to model animal habitat, applications on the plant habitat modeling are extensively in the recent literature^41–43^. Geospatial environmental data (topography, climate, vegetation type) are usually used as predictors for modeling the habitat suitability^42^.

**Figure 4:**
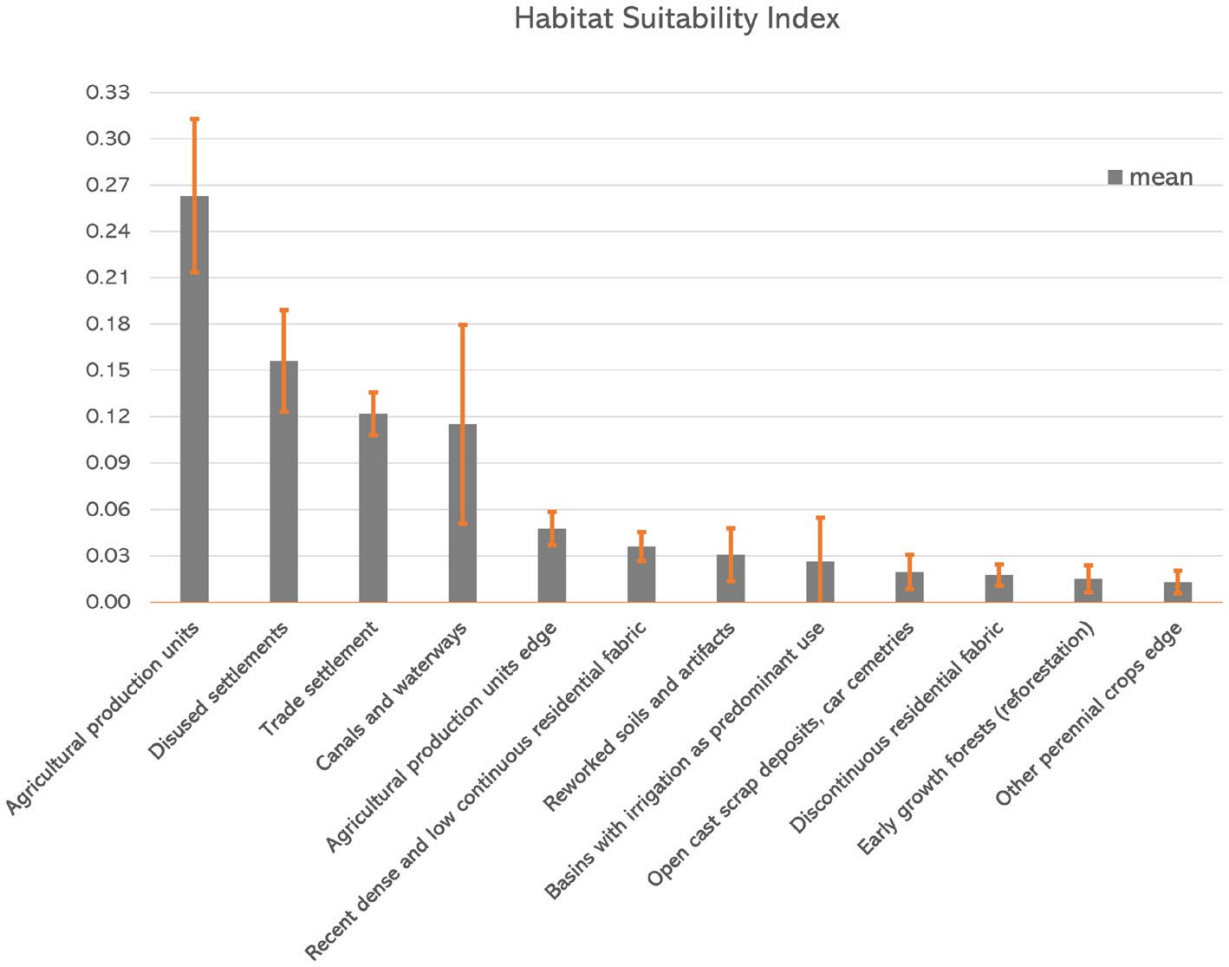
Estimated mean and standard deviation of habitat suitability index of *A. altissima* for LU classes in the analyzed area.

Due to the lack of data, in this paper we give a rough estimate of the habitat suitability, starting from the already extracted presence map in 2012 and the Land Use (LU) map available from the Regione Puglia repository (http://www.sit.puglia.it/portal/portale_cartografie_tecniche_tematiche/Cartografie%20Tematiche/UDS), for the same year. Due to the propensity of the plant to grow along the edges of the woods, along roadsides, rows and in the openings of the woods, we extended the 46 LU classes in a Corine Land Cover-like taxonomy with ten more classes defining the edges of: deciduous forests, olive groves, orchards and small fruit farms, early growth forests, mixed coniferous and deciduous forests, coniferous forests, agricultural production units, vineyards, cultivation and complex systems and other perennial crops. All the analyzed LU classes are listed in Fig. 4, togheter with the HSI estimated valued.

**Figure 5:**
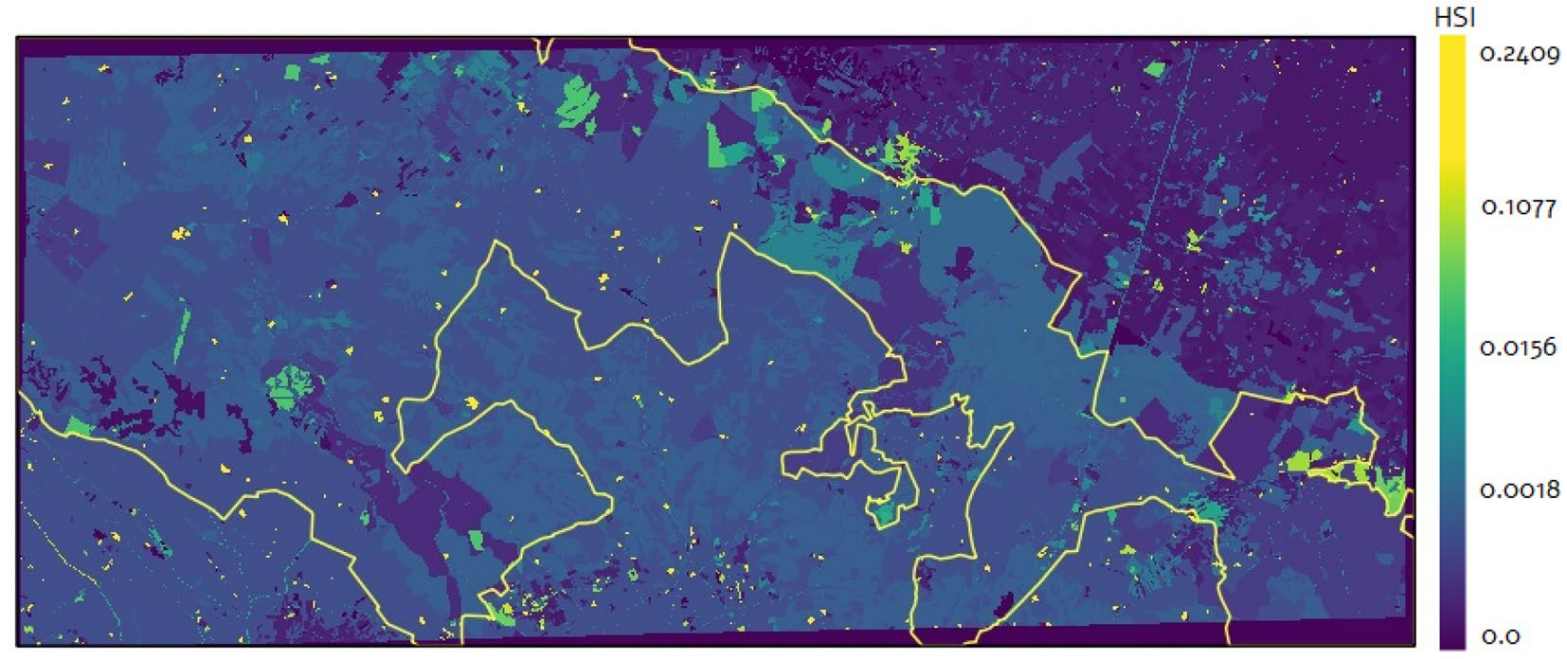
Habitat suitability map in the study area. Yellow line: boundary of the Alta Murgia National Park. Map obtained by RStudio, version 2023.03.0 (https://cran.rstudio.com/) and QGIS 3.24 (https://qgis.org/en/site/).

We produced the HSI map with a spatial resolution of 20 m: the spatial degradation was preferred for computational purposes. The HSI corresponding to each LU class has been calculated as proportional to the frequency of occurrence of that LU class in the surrounding of each pixel where the presence of *A. altissima* is detected. The rationale behind is that the more a LU class appears nearby an *A. altissima* tree the more it is suitable to host the invasive species. As we only have a single presence map of *A. altissima*, we used the same dataset for both estimation and evaluation. In particular, we split the map into two horizontal strips and apply the estimation procedure to the upper one. Then, the fitted HSI values were compared with the occurrences of the plant in the lower strip of the map.

To have an unbiased HSI calculation, we applied a subsampling method, specifically we split the initial map in $k=5$ chunks and we computed the suitability map on the remaining $k-1$ chunks; then, we repeated this procedure for *k* times by excluding the kth chunk at each iteration. The final map was obtained as the mean of the suitability values obtained in each iteration. In Fig. 4, we have represented the bar-plot with the mean and standard deviation of the HSI only for the most suitable habitats, omitting those that were not suitable at all. The maximum value of HSI was 0.262638 corresponding to the *agricultural production unit* LU class. The estimated mean and standard deviation of the habitat suitability indices of *A. altissima* in each LU class are shown in Fig. 4. The mean values were projected over the LU map to obtain the HSI map in Fig. 5.

### Model validation

For assessing the predictive power of the applied procedure for estimating the HSI values, based on the presence map of *A. altissima*, we used the Boyce index^44^. The index quantifies the ability of the estimated HSI map in predicting the presence of the plant, given a set of evaluation points. The method consists in partitioning the values of the habitat suitability into a number *k* of classes and calculating the predicted frequency in each class. Then, this frequency is compared with the expected one, i.e. the expected frequency of a random distribution of presence points. The two frequencies are defined as follows:

$$\begin{aligned} P_i= \frac{p_i}{\sum _{j=1 }^ {k} p_j } \end{aligned}$$

is the predicted frequency in class *i* and the expected frequency is calculated as the relative area covered by each class i , with $i =1 \dots k$

$$\begin{aligned} E_i= \frac{a_i}{\sum _{j=1 }^ {k}a_j }\end{aligned}$$

where $a_i$ is simply the number of cells covered by the i-th habitat suitability class. The ratio of the predicted-to-expected frequency $F_i= P_i/E_i$ provides a good indication of the capability of a model of simulating the real habitat suitability. Indeed, we do expect that a good model exhibits a monotonic increase of $F_i$ with the class it refers to. The Boyce index is then defined as the Spearman rank correlation coefficient between $F_i$ and *i*. The values of the index vary from $-1$ to 1 with positive values indicating that predictions correlate with the data, whereas the negative values characterize the absence of correlation. Values close to zero tell us that the model is not different from a random choice. In this paper we used a continuous version of the Boyce index, introduced in^27,44^, where the fixed classes are substituted by a moving window of a given size. The continuous Boyce index has been developed to solve the issue of the sensitivity of the index to the fixed number of classes and is available as a R code (https://github.com/jmrmcode/contboyceindex) which has been used in the present paper to evaluate the predictive ability of our model. As evidenced in the previous section, to perform the evaluation we divided the original presence and land cover maps in two parts, the upper one used to build our habitat suitability model, the lower one to evaluate the predictive ability. On that last portion of the map we measured the continuous Boyce index, by associating at each land cover class of the map the habitat suitability previously estimated and then calculating the predicted-to expected ratio. The evaluation has been repeated at different resolutions of the original land cover map, starting from 2 m, with the highest values of the Boyce index corresponding to the highest resolution and close to 1. Since different resolutions generate different distributions of land cover classes the degradation of the index is somehow expected. However, the predictive ability of the model is preserved in a considerable range of decreasing resolutions. E.g., at 20 m, ten times more than the original one, which is the resolution used throughout the paper to perform the simulations, we obtain a continuous Boyce index value of 0.71 which still indicates a good predictive ability of the model in spite of the bias generated by the higher resolution.

## The spatiotemporal optimal control model

The formulation of the optimal control problem is based on a PDE reaction-diffusion model with logistic growth, that includes a control term that has Holling-II type behavior and a budget constraint^11^. The invasive species abundance, u(**x**, t), at position x and time t is given by:

1 $$\begin{aligned} \frac{\partial u}{\partial t}(\textbf{x},t) = D\,\Delta u(\textbf{x},t) + r\,u(\textbf{x},t)\left( \rho (\textbf{x})\,-\frac{u(\textbf{x},t)}{k}\,\right) -\frac{\mu \,u(\textbf{x},t)\,E(\textbf{x},t)}{1+\tau \,\mu \,u(\textbf{x},t)} \end{aligned}$$

The first term on the right-hand side describes collective motion of randomly moving individuals depending on a coefficient *D*, the diffusivity, which governs how quickly the species disperses. The second term on the right-hand side is a logistic population growth term, where the coefficient *r* is the intrinsic growth rate of the population and *k* is the carrying capacity. The term $\rho (\textbf{x})$, bounded between 0 and 1, represents the habitat suitability function which modulates the carrying capacity *k* according to the suitability of the land cover to the growth of the invasive species. The final term represents the species mortality due to a control effort $E(\textbf{x},t)$, which is restricted to non-negative values. The control action on *u* is modeled by a Holling II-type function $\mu u/(1+ \tau \mu u)$ , where $\mathrm {\mu }$ is the harvesting rate per population density unit, due to control. The average time spent for the harvesting of a population item is represented by the positive coefficient $\tau$. In the Supplementary Information, we reported the estimated parameters for *A. altissima* growth and eradication.

The goal that we consider is to minimize the environmental damage over time at the minimum cost, in terms of the resources allocated to the species harvesting. A penalty term is added to take into account the budget constraint $E(\textbf{x},t)\le B$. As a result we build the penalized objective function as follows

2 $$\begin{aligned} \mathfrak {J}\left( E\right)=\, & \displaystyle \alpha \int _{0}^T \, e^{-\delta t} \left( \int _{\Omega } E^2\left( \textbf{x},t\right) d\textbf{x}\right) \, dt + \beta \int _{0}^T \, e^{-\delta t} \left( \int _{\Omega }\frac{E^3\left( \textbf{x},t\right) }{B^3} d\textbf{x}\right) dt \nonumber \\{} & {} + \displaystyle \gamma \,\int _{0}^T \, e^{-\delta t} \int _{\Omega }u\left( \textbf{x},t\right) d\textbf{x} \, dt + \theta \, e^{-\delta \,T}\, \int _{\Omega }u\left( \textbf{x},T\right) d\textbf{x}, \end{aligned}$$

where $\gamma$ represents the cost due to the environmental damage, $\theta$ is a weight for the final population density, $0<\delta <1$ is the discount factor. The term $\beta \, \int _{\Omega \times [0,T]}e^{-\delta t}\left( \frac{E\left( \textbf{x},t\right) }{B}\right) ^{3}\,d\textbf{x}\,dt ,$ represents the penalty term due to the budget constraint.

The model parameters were estimated on the basis of the expert knowledge of the plant and of the control program applied in 2014–2019 Alta Murgia National Park within the Alta Murgia Project. The parameter estimation and the numerical procedure for the approximation of the system, as well as the description of the codes written in R open source language, are given in the Supplementary Information.

### Diffusion over 10 years

**Figure 6:**
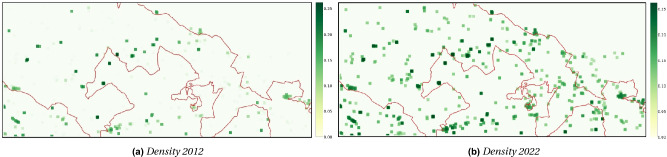
Dynamics of the diffusion model. Increase of density in 10 years, starting from 2012 until 2022. Maps were calculated by multiplying the presence by suitability and applying a Gaussian filter to highlight the changes.

We firstly run the PDE model (1) over a period of 10 years, from 2012 to 2022, with a spatial resolution of 20 m. In this way we show the evolution of the invasive plant due to the effects of both growth and diffusion. The initial presence map (Fig. 2 in Supplementary Information) was multiplied by the habitat suitability map in Fig. 5 in order to obtain the initial density map in Fig. 6a. Assuming that no control action was applied for the whole analyzed period, i.e. $E(\textbf{x},t)=0$ for each cell $\textbf{x}$ and $t\in [2012,2022]$, model (1) predicts the growth and diffusion of *A. altissima* in the area, whose final state is depicted in Fig. 6b. Note that for the purposes of visualization, we applied a Gaussian filter to aggregate the results of clusters of cells with non-zero mean density.

#### Uncertainty quantification with respect to HSI values

Arbitrary Polynomial Chaos^31^ (aPC) was used to quantify the effect of uncertainties in the habitat suitability scores on the predictions of the model. aPC provides a spectral representation of uncertainty, with the density represented by the expansion:

3 $$\begin{aligned} u(\varvec{x}, t|\varvec{\xi })=\sum _{k=0}^P \varvec{\alpha }_k(\varvec{x},t)\Psi _k(\varvec{\xi }), \end{aligned}$$

where $\varvec{\xi }\in \mathbb {R}^{n_u}$ collects the $n_u$ uncertain habitat suitability indices, $\varvec{\alpha }(\varvec{x},t)\subseteq \mathbb {R}^{P+1}$ is a set of deterministic coefficients and $\Psi$ a multivariate orthogonal polynomial, itself a product of a set of uni-variate, orthogonal polynomials, $\psi$, with:

4 $$\begin{aligned} \Psi _k(\varvec{\xi })=\prod _{i=1}^{n_u}\psi _{I_{k,i}}(\varvec{\xi }_i), \;\; k=0,\dots , P. \end{aligned}$$

$I=(I_{k,i})$ is a $(P+1) \times n_u$ index matrix, with the rows denoting the corresponding orders of the uni-variate polynomials for each term in the expansion. *P*, the number of terms in the expansion, is a function of $n_u$ and the degree of the polynomial basis, denoted *p*:

5 $$\begin{aligned} P+1=\frac{(n_u+p)!}{n_u!p!}. \end{aligned}$$

Given samples of the joint density, $f(\varvec{\xi })$, the aPC formulation used here computes an optimal multi-variate polynomial basis using the statistical moments of these samples. Note that the Polynomial Chaos formulation assumes that the uncertain habitat suitability indices are statistically independent, i.e. $f(\varvec{\xi }$)=$\prod _{k=1}^{n_u}f_k(\varvec{\xi }_k)$, where $f_k$ is the Probability Density Function (PDF) for the *k*th uncertain habitat suitability index. Here we focus on uncertain HSI values for four common land cover classes: agricultural production units; bushes and shrubs; areas with natural recolonisation; natural pastures, grasslands, uncultivated. Assumed Gaussian distributions for these parameters were used in the uncertainty analysis.

**Figure 7:**
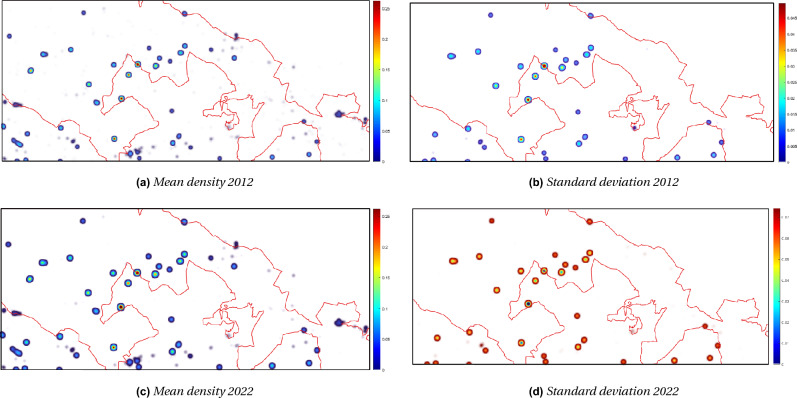
On the left, the density mean at time 2012 (**a**) and 2022 (**c**), generated from the nine HSI maps sampled with the aPC algorithm; on the right, the uncertainty measured by the standard deviation at the initial (**b**) and final (**d**) time.

Determining the coefficients of the expansion through a least squares fitting requires at least $P+1$ model evaluations. As can be seen from (5), *P* scales rapidly with the number of uncertain parameters and polynomial degree. This phenomenon is referred to as the *curse of dimensionality*. To mitigate this effect, the aPC formulation used here employs Smolyak’s algorithm^45^ to assemble a sparse sampling grid on which to evaluate the model. For degree $p=2$, a sparse sampling grid containing nine points was assembled using the collocation points of the PDFs and Smolyak’s algorithm. The co-ordinates of these sampling points are tabulated in Table 2 in the Supplementrary information. Denoting the *i*th of the $n_\theta =9$ sampling points as $\varvec{\theta }^{(i)}\in \mathbb {R}^4$, the weights in (3) may be computed through^8^:

6 $$\begin{aligned} \varvec{\alpha }_k(\varvec{x},t)=\frac{\sum _{i=1}^{n_\theta }\varvec{w}_i u(\varvec{x},t|\varvec{\theta }^{(i)})\Psi _k(\varvec{\theta }^{(i)})}{\sum _{i=1}^{n_\theta }\varvec{w}_i \Psi _k(\varvec{\theta }^{(i)})}, \end{aligned}$$

where $\varvec{w}\in \mathbb {R}^{n_\theta }$ represents the weights from Smolyak’s algorithm and $u(\varvec{x},t|\varvec{\theta }^{(i)})$ an evaluation of the model, conditioned on the HSI values in $\varvec{\theta }^{(i)}$. The PDE model (1) with no control action ($E(\textbf{x},t)=0$) was evaluated at each of the nine points of the grid, over a period of 10 year resolution with a spatial resolution of 20 m. The nine initial density maps at 2012 were obtained by multiplying the presence map (0/1) by the estimated HSI values at the collocation points. Then, model (1) was run for each of those maps and the mean and standard deviation (sd) values of the predicted density values were derived on the whole domain at different times. Figure 7 shows the mean and sd maps at the initial and final times.

### The control program

**Figure 8:**
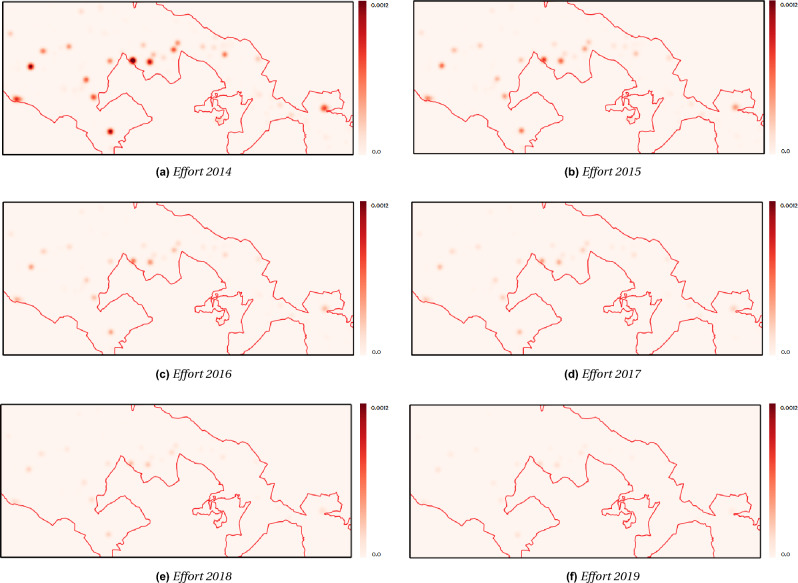
Maps of the control effort variable showcasing the results of utilizing the optimal control model (1)–(2) to devise an optimal resource allocation strategy for eradicating the plant within Alta Murgia National Park from 2014 (**a**) to 2019 (**f**).

Starting with the initial density map in Fig. 6a, we ran the PDE model (1) for 2 years to obtain the density map at 2014. Next, we employed the optimal control model (1)–(2) to determine the best resource allocation strategy for eradicating the plant within the Alta Murgia National Park from 2014 to 2019. The resulting control maps in Fig. 8 illustrate the optimal allocation of efforts over time and space for the most effective removal strategy.

**Figure 9:**
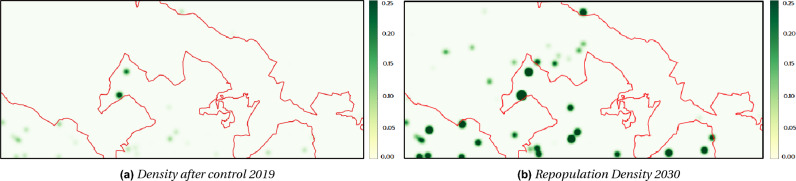
(**a**) Density map illustrating the nearly complete eradication of the plant within the park’s boundaries by 2019, subsequent to the implementation of the control program. (**b**) Density map depicting the post-project repopulation density projected for the year 2030, as determined by the PDE model (1) simulation conducted without any control actions from 2019 to 2030.

By the end of the program in 2019, the plant had been nearly completely eradicated within the park’s boundaries, as shown in the density map in Fig. 9a. As expected, the dynamic evolution of the model demonstrates a steady decrease in plant density, leading to a significant reduction within the park. The control maps in Fig. 8 visually depict this trend, with the red color indicating density fading as more plants are removed. The density values within the study area by 2019 are very low, indicating the near absence of plants (Fig. 9a).

We further simulated the repopulation process after the conclusion of the project by running the PDE model (1) without any control action from 2019 to 2030. The density prediction of the plant for 2030 is depicted in Fig. 9b. The resulting density maps (Fig. 9a,b) visually illustrate the regrowth of *A. altissima* during the years leading up to 2030. As anticipated, the plant reestablishes within the Alta Murgia National Park and shows an increase outside the perimeter where the eradication program was not implemented.

## Discussion

The Italian National Biodiversity Future Centre (NBFC)^6^ is one of the National Centers founded in 2023 under the Italian National Recovery and Resilience Plan, which aims to conserve, restore and limiting the loss of biodiversity in Italy. Assessing and monitoring biodiversity and its evolution and to plan protection and conservation interventions to protect native species at risk of extinction because of IAS spread, are some of its specific tasks. Development and implementation of model-based decision support systems are a key asset for effective control programmes for IAS.

Addressing the complex issue of IAS management is an ongoing challenge. The scale of the problem, with an ever-growing number of invasive species and their territorial expansion, leads to diverse impacts on invaded or vulnerable areas. Different management options, such as prevention, containment, control, or eradication, must be carefully considered based on specific circumstances. Due to the magnitude of the IAS problem and the limited resources available for their control, careful planning is crucial. Cost-effective solutions that minimize negative impacts on socio-ecological systems require in-depth analysis of the extent of invasion and predictions of its evolution over time and space.

The issue of management costs compared to damages caused by IAS is gaining attention in the scientific community. IAS exert substantial economic and social consequences, resulting in property devaluation and decreased agricultural productivity. To optimize cost-effectiveness, it is imperative to invest in timely and proactive management measures^46^. Research shows invasive species cause damages ten times higher than management costs, with damage costs increasing rapidly^20^. However, insufficient implementation of international agreements is evident from rising global damage costs^20^. A recent paper discusses economic benefits of managing invasive species, comparing avoided costs with and without intervention, including control costs and benefits forgone^47^.

The description of the spatiotemporal dynamics of plant invasive species is an important tool that allows an understanding of natural phenomena and how they should be managed in the short and long term to preserve biodiversity and improve the sustainability and conservation of nature. For this purpose, in this work, a PDE optimal control model with penalty term (1)–(2) was implemented to automatically predict the density of vegetative IAS and to determine the best allocation strategy for the control of the plant. We focused our attention on the case study of the invasive species *A. altissima*, a pervasive invasive plant species in the Alta Murgia National Park, a Natura200 site in the Southern Italy, one of the pilot site of Task 4.3 of the NBFC. *A. altissima* has been chosen as target IAS species because has quickly spread in the Alta Murgia National Park, causing serious direct and indirect damages to ecosystems and natural habitat loss and degradation. *A. altissima* harms the ecological balance of the park threatening the its native biodiversity. As only an active management can ensure the conservation of the wild flora and fauna species of the park, the European Commission funded the Life Alta Murgia Project to eradicate of this invasive exotic tree species. We made use of the insights gained from the Life Alta Murgia Project to evaluate the costs involved in eradication. Our objective was to identify the most efficient allocation strategy for utilizing the available budget for eradication efforts. To estimate the costs, we accounted for the daily expenses of a single eradication team, encompassing herbicide costs and the required resources for clearing specific areas. For a more comprehensive and detailed cost estimation, please refer to the Supplementary Information.

As a first analysis, we applied Eq. (1) without the control term to describe the diffusion dynamics of the species *A. altissima* in the Alta Murgia National Park. Using the presence map for the year 2012 and the estimated HSI map at a resolution of 20 m, we observed an increase in density around the spatial points where the species was present in the PDE model at 2022 (Fig. 6). The Boyce index demonstrated good predictive capability of the HSI estimation based on the presence map of the plant, despite the resolution bias. Additionally, the aPC technique quantified the uncertainty effect on the predicted map at 2022. The density map revealed increased mean density between 2012 and 2022, with uncertainty concentrated on the edge of zones with high mean density, represented by red rings in Fig. 7d. The analysis indicates a uniform distribution around the source, suggesting equal diffusion in all directions. Over time, the variation in density spreads from the center towards the edges of the cell, with a “cone of uncertainty” represented by a confidence interval. Detailed results are provided in the Supplementary Information, including changes in mean density and uncertainty assessment for individual cells.

A detailed perspective on a cluster of cells shows that the distribution around the source is uniform. This uniformity indicates that there are no natural structures in the region influencing or imposing a specific preferred direction for diffusion. In other words, particles or substances diffuse equally in all directions without any bias. When examining the standard deviation, it becomes evident that the variation is initially higher around the center of the cell. As time progresses, this variation gradually spreads towards the edges of the cell. This observation suggests that at the beginning of the process, the dispersion of particles is more concentrated near the center. However, over time, it becomes more evenly distributed throughout the cell. Additional details of this analysis are presented in the Supplementary Information. The analysis therein includes changes in mean density and introduces the concept of the “cone of uncertainty,” represented by a $2\sigma$ confidence interval, for a single cell. This uncertainty takes into account the variability and confidence level of the data.

The analysis suggests that the parameters that most influence the dynamics are the HSI values, which indicates how close the local environment is to the optimal growth conditions of the species. A possible extension of this work would be to analyze how the results change with respect of the HSI. The estimation of the habitat suitability index indirectly incorporates human influence by utilizing the LCCS-FAO land cover taxonomy, which encompasses both natural and anthropogenic classes and provides a high level of thematic detail, with potentially infinite classes. The prevailing method for estimating habitat suitability is typically based on correlating the presence of the species with biophysical environmental conditions^48^, which often overlooks the role of human influence and requires a substantial amount of data. The underlying rationale of our approach is to account for the relevance of human influence through a land cover map extracted from a very high resolution image and classified according to FAO-LCCS taxonomy. While land cover maps are inherently linked to biophysical variables, they also reflect the impact of human activities. As an example, Fig. 4 illustrates that our land cover map includes classes such as “disused settlements” that have a notable tree presence. Roads, settlements, urban areas, as well as the existence of canals and other linear features, such as agricultural field edges, are all considered and weighted within the habitat suitability index. Given the role of HSI in the model, and the way the model operates make it unnecessary to explicitly incorporate distance or density measurements of artificial landscape components.

As a second analysis, the model (1)–(2) was employed to find the best resources allocation strategy for the eradication program of the plant in the years 2014–2019. The simulations show that the model is well suited for the study of invasive species. It is both able of describing the dynamics of diffusion and providing predictions on the effort to be considered to contain the spread of the species. Starting from the initial presence density of *A. altissima*, obtained from an automatic data-driven classification approach applied on satellite images, our simulations are able to suggest to a park manager with limited budged resources *where and when* allocating the control effort in terms of number of teams of person (able to eradicate approximately 1000 plant a day), for removing as much as possible the alien plants within the temporal window of the management action.

The work presented here combines remote sensing to estimate habitat suitability with spatiotemporal optimal control into a single workflow. Some authors also use very high-resolution WorldView-2 imagery to develop maps of invasive species presence, compared to orthophotos from unmanned aerial vehicles for training and validation^49^. There is also a body of work that links remote sensing directly to management using unmanned aerial systems^50^. This line of investigation focuses on the potential of utilizing artificial intelligence technologies in agriculture, specifically for effective weed management, but it does not link remote sensing to optimisation routines. On the other hand, some works build on optimal control analyses developed to optimize spatiotemporal resource allocation, but where habitat suitability is already known^7,51^. Another recent paper focuses on predicting the spatiotemporal spread of invasive species using elevation-dependent habitat suitability and arbitrary Polynomial Chaos method is applied to assess parameter uncertainty and enhance the accuracy of predictions^8^. However, the paper does not incorporate any control measures for managing the invasive species. Finally, other work focuses on the issue of imperfect detection and misclassification within spatiotemporal invasive species management and highlights the opportunities available by combining optimisation techniques with remote sensing^52^.

From a modeling perspective, IAS removal has been approached using different strategies in previous studies, such as mass action interactions and constant rate of removal^53,54^. However, both of these methods have their drawbacks. Mass action interactions, which assume that the rate of removal is proportional to the control effort multiplied by abundance, become unrealistic when dealing with high abundances of invasive species. This unrealistic behavior occurs because the removal rate can become exceedingly large for very large populations of invaders. On the other hand, constant removal models, where the rate of removal is simply proportional to the control effort, have their limitations too. These models are not suitable for small populations of invasive species because the removal rate does not decrease as the population declines. This lack of decrease in the removal rate does not account for the increased search time required to find individual invasive species as their population becomes rare. To achieve a more balanced and realistic representation of invasive species removal, we used a Holling-II function response in Eq. (1), to describe the effect of the control action on the population dynamics^7, 11^. By combining the features of mass action at low abundances and saturation to constant removal at high abundances, this approach enhances our comprehension of how control efforts influence population levels. The incorporation of the Holling-II function response leads to a more accurate representation of invasive species removal dynamics. This improvement aids decision-makers and managers in assessing the potential effectiveness of various control strategies and optimally allocating resources for eradicating or containing invasive species.

This work stands out from others in the field due to its combination of expert knowledge, remote sensing techniques, and spatiotemporal optimal control. This integration creates a comprehensive and robust approach to invasive species management and control. By leveraging expert knowledge, the work ensures that decision-making is informed by deep domain expertise. The utilization of remote sensing techniques enables a more accurate understanding of the distribution of invasive species. Lastly, the incorporation of spatiotemporal optimal control optimizes resource allocation, leading to more efficient and effective management strategies. This integrated framework enhances the overall precision and effectiveness of invasive species management efforts.

Our approach to IAS management could act as a decision support system for managers, providing them a tool to tackle the complexities of invasive species control effectively. We provide a robust and data-driven framework to make informed decisions and take targeted actions. One of the key advantages of the model is its adaptability, allowing it to be tailored to suit the specific characteristics and behaviors of various invasive species and ecosystems. By adjusting the model’s parameters, such as biological traits, diffusion rates, and growth coefficients, managers can obtain accurate representations of the invasive species they are dealing with, enhancing the precision of management strategies. The model helps optimize the allocation of these resources by predicting the density of invasive species in different areas and suggesting control efforts accordingly. By focusing control efforts where they are most needed, managers could achieve more significant impacts with the resources at hand, ultimately leading to more cost-effective and efficient management strategies. A critical aspect of our model is its capability for early detection and monitoring. After control efforts have been implemented, the model can be used to monitor the spread of invasive species over time. By proactively addressing potential re-infestation, managers can take timely action and prevent the resurgence of invasive species.

Since the eradication program takes place in a protected area that is geographically limited but has no physical barriers at the edges, once the control action ends, the invasion of the species starts again from the boundaries of the park close to the private areas where the eradication is not allowed. Our simulations in case of absence of control from 2019 to 2022 are capable of making an early detection of re-infestation as to suggest to managers *where and when* concentrating their efforts in case of additional financial resources. Implementing control measures beyond the national park’s borders, where feasible, could prove to be a valuable strategy in mitigating re-invasion. In this study, our focus was on testing and validating spatiotemporal control within the protected area, where active eradication efforts were underway, and relevant data were accessible. However, by incorporating data from neighboring areas, the model could demonstrate the effectiveness of controlling the invasive species in adjacent regions to reduce its influx into the protected area. Understanding the implications of implementing control measures beyond the park’s borders is crucial for designing a comprehensive and efficient eradication program. In this study we utilized in-field reference data within the park boundaries, resulting in a lack of validation information for the surrounding area. Nevertheless, once the entire process is validated, it can be applied wherever field data are available. For future research and modeling endeavors, there is a valuable opportunity to explore the potential benefits of adopting a broader approach to invasive species control, encompassing both the protected area and its neighboring regions. Such investigations could offer valuable insights to policymakers and managers, aiding them in developing a more integrated and sustainable management strategy for invasive species eradication and conservation efforts.

Management actions aimed to eradicate and control IAS in protected areas whose borders have no physical barriers cannot be successful on the long time horizon. Consequently, the models that simulate these actions give us an optimal solution for the ongoing use of financial resources to strive for the absence of the IAS. A future development of our tool could deal with spatial domains in which there are real physical barriers such as, for example, the alpine lakes of the Gran Paradiso National Park^55^, another pilot site of NBFC. Our tool could also be useful for supporting decisions for containment of invasive alien fish and thereby mitigate their environmental impacts. To do so, we would need to modify the underlying optimality system, to take into account the barrier-type boundary conditions. Other potential developments include wind or current effects modeled by transport terms^26^, whose effects are neglected in this first version. Finally, new approaches based on ecological networks both for the containment of IAS and for simulating the effects of the re-population of native species will be the object of our study in the next future.

## Conclusions

Addressing the complexity of IAS management requires a comprehensive approach, incorporating expert knowledge, remote sensing techniques, and spatiotemporal optimal control. This integrated framework enables more accurate and data-driven decision-making, allowing for effective resource allocation and targeted control efforts. By optimizing the allocation of resources, decision-makers can achieve more significant impacts while minimizing negative effects on socio-ecological systems.

The implementation of a PDE optimal control model with a penalty term, combined with habitat suitability index (HSI) estimation from remote sensing data, has proved to be a valuable tool for predicting the density of invasive species and suggesting optimal allocation strategies for eradication programs. This approach is adaptable and transferable, making it applicable to different invasive species and ecosystems. The model’s optimization process considers factors such as timing and location of control actions, leading to more efficient and cost-effective management strategies. Furthermore, the model allows for early detection and monitoring of re-infestation, enabling managers to take timely action and prevent the resurgence of invasive species.

While the eradication programs in protected areas without physical barriers may not achieve long-term success, the model can still provide valuable insights into resource allocation and containment efforts. Future developments of the model could incorporate physical barriers and address other challenges, such as wind or current effects, to enhance its applicability in different scenarios.

### Supplementary Information

Supplementary Information.

## Data Availability

The workflow “Ailanthus Workflow—Combining Modeling and remote sensing techniques to monitor and control the spread of invasive species:the case of Ailanthus altissima” was developed within the Internal Joint Initiative (IJI) of LifeWatch ERIC https://metadatacatalogue.lifewatch.eu/srv/api/records/66744693-cfc7-4901-9838-618e18bb17af. The same codes were ported to the ECOPOTENTIAL Virtual Lab platform (VLab) at https://vlab.geodab.org/. The R^®^ codes COINS.R (COntrol of INvasive Species) and HabSuit.R, for the modelistic implementation and the HSI estimation, respectively, are available from https://github.com/CnrIacBaGit/COINSvlabrepo.
