# Supplementary material for: Using remote sensing data within an optimal spatiotemporal model for invasive plant management: the case of Ailanthus altissima in the Alta Murgia National Park
Source: Sci Rep. 2023 Sep 4;13:14587. doi: 10.1038/s41598-023-41607-2 (PMC10477239; doi:10.1038/s41598-023-41607-2)
Supplement: Supplementary file 1 — Supplementary Information. [file 41598_2023_41607_MOESM1_ESM.pdf]

Supplementary Information to "Optimal spatiotemporal model for invasive plant management: the case of *Ailanthus altissima* in the Alta Murgia National Park"

Christopher M. Baker, Palma Blonda, Francesca Casella, Fasma Diele, Carmela Marangi, Angela Martiradonna, Francesco Montomoli, Nick Pepper, Cristiano Tamborrino, and Cristina Tarantino

In this document, we provide some additional information useful to complement the paper "Optimal spatiotemporal model for invasive plant management: the case of *Ailanthus altissima* in the Alta Murgia National Park". As first, we provide the details regarding the classification algorithm for the plant detection. We also provide tables for the uncertainty quantification analysis. Finally, we estimate all the parameters used in the main document, based on the expert knowledge of the plant and the Life Alta Murgia Project (LIFE12 BIO/IT/000213, `https://www.ispacnr.it/progetto-life-alta-murgia/`) control program applied in 2014-2019. Moreover, we describe the numerical procedure for the model approximation and show the numerical codes written in R open source language.

Two-stages classification algorithm for plant selection

Figure 1 schematises the hybrid two-stage classification algorithm adopted to produce the *A. altissima* mapping.

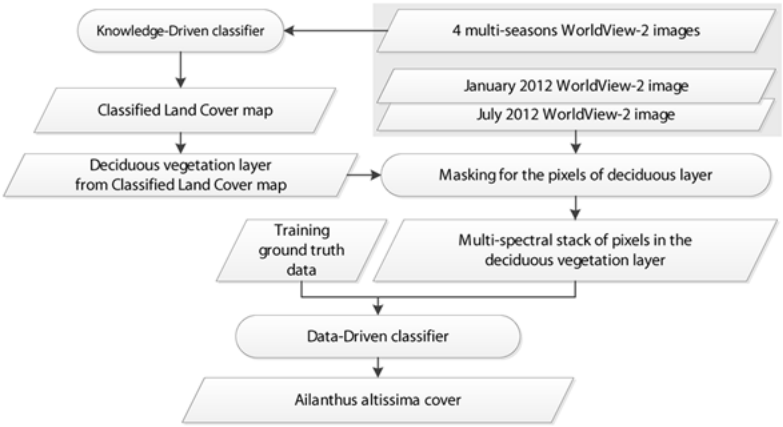

Figure 1. Two-stages classification algorithm. On the left, object-oriented, knowledge-driven first stage; on the right, pixel-oriented, data-driven second stage.

In Figure 2 we show the binary map of the initial presence (2012) of *A. altissima* in the analyzed area, obtained by applying the algorithm. The pixels in green color (value 1) indicate the *A. altissima* presence. Due to the 2 m spatial resolution of the map, for display reasons, we need to apply a Gaussian filter to highlight those pixels corresponding to the presence.

Uncertainty quantification analysis

In this part we provide the parameters for estimation of the uncertain HSI values. As reported in the main document we have considered only four common land cover classes: agricultural production units; bushes and shrubs; areas with natural recolonisation; natural pastures, grasslands, uncultivated. Table 1 reports the mean and standard deviations of the HSI in the considered LC classes. Table 2 reports the values of these sampling points.

LC class	Mean	Standard deviation
Agricultural production units (APU)	0.262974741	0.049765945
Bushes and shrubs (BS)	0.017000519	0.004564249
Areas with natural recolonisation (NR)	0.008851223	0.004236427
Natural pastures, grasslands, uncultivated (NPGU)	0.001990302	0.000212548

Table 1. Mean and standard deviation values for the HSI gaussian distributions in the four selected LC classes.

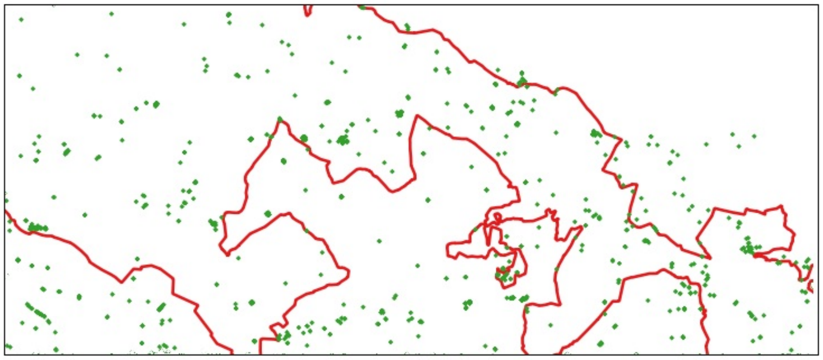

Figure 2. Presence map of *A. altissima* in 2012, with highlighted green points in the selected sub-region of Alta Murgia National Park. Map obtained by RStudio, version 2023.03.0 (https://cran.rstudio.com/) and QGIS 3.24 (https://qgis.org/en/site/).

APU	BS	NR	NPGU
0.21315795	0.017008693	0.008855831	0.001990389
0.262992132	0.012450431	0.008855831	0.001990389
0.262992132	0.017008693	0.004616853	0.001990389
0.262992132	0.017008693	0.008855831	0.001778036
0.262992132	0.017008693	0.008855831	0.001990389
0.262992132	0.017008693	0.008855831	0.00220322
0.262992132	0.017008693	0.013085945	0.001990389
0.262992132	0.021571965	0.008855831	0.001990389
0.312622103	0.017008693	0.008855831	0.001990389

Table 2. The nine locations in the sparse sampling grid at which the model was evaluated.

**Symbol**	**Variable**	**Ecological meaning**	**Dimension**
u	Plant density	#plant in the unitary area	km^{-2}
E	Effort density	# team in the unitary area	km^{-2}

Table 3. Model's variables and related dimensions.

Figure 3 shows mean and standard deviation for a single cell over the ten year period. It provides a granular view, showing the mean and sd for a single cluster of cells with non-zero mean density. In Figure 3g the location of this cluster is highlighted in red. In Figures 3a-3c uniform distribution around the source can be observed. This is because there are no natural structures in the region that impose a preferred direction for diffusion. For the standard deviation, plotted in Figures 3d-3f, the greater variation is associated to the center of the cell and over time spreads towards the edges. Figure 3g illustrates the change in mean density and associated cone of uncertainty (a 2σ confidence interval) for a single cell. The location of this cell is highlighted in red in Figures 3h.

Model's parameters selection

The model consists of the following equation

$$\frac{\partial u}{\partial t}(\mathbf{x},t) - D\Delta u(\mathbf{x},t) = r\, u(\mathbf{x},t)\left(\rho(\mathbf{x}) - \frac{u(\mathbf{x},t)}{k}\right) - \frac{\mu\, u(\mathbf{x},t) E(\mathbf{x},t)}{1+\tau\mu\, u(\mathbf{x},t)} \tag{S.1}$$

where u and E represent the plant densities and the effort density, respectively, and are measured in km^{-2} (see Table 3). We assume that an item of control corresponds to a team of 4 persons. The model parameters have been selected as follows:

- r: *A. altissima* growth rate[1]. The *A. altissima* growth rate is set at $r = 1.92$ year^{-1}.

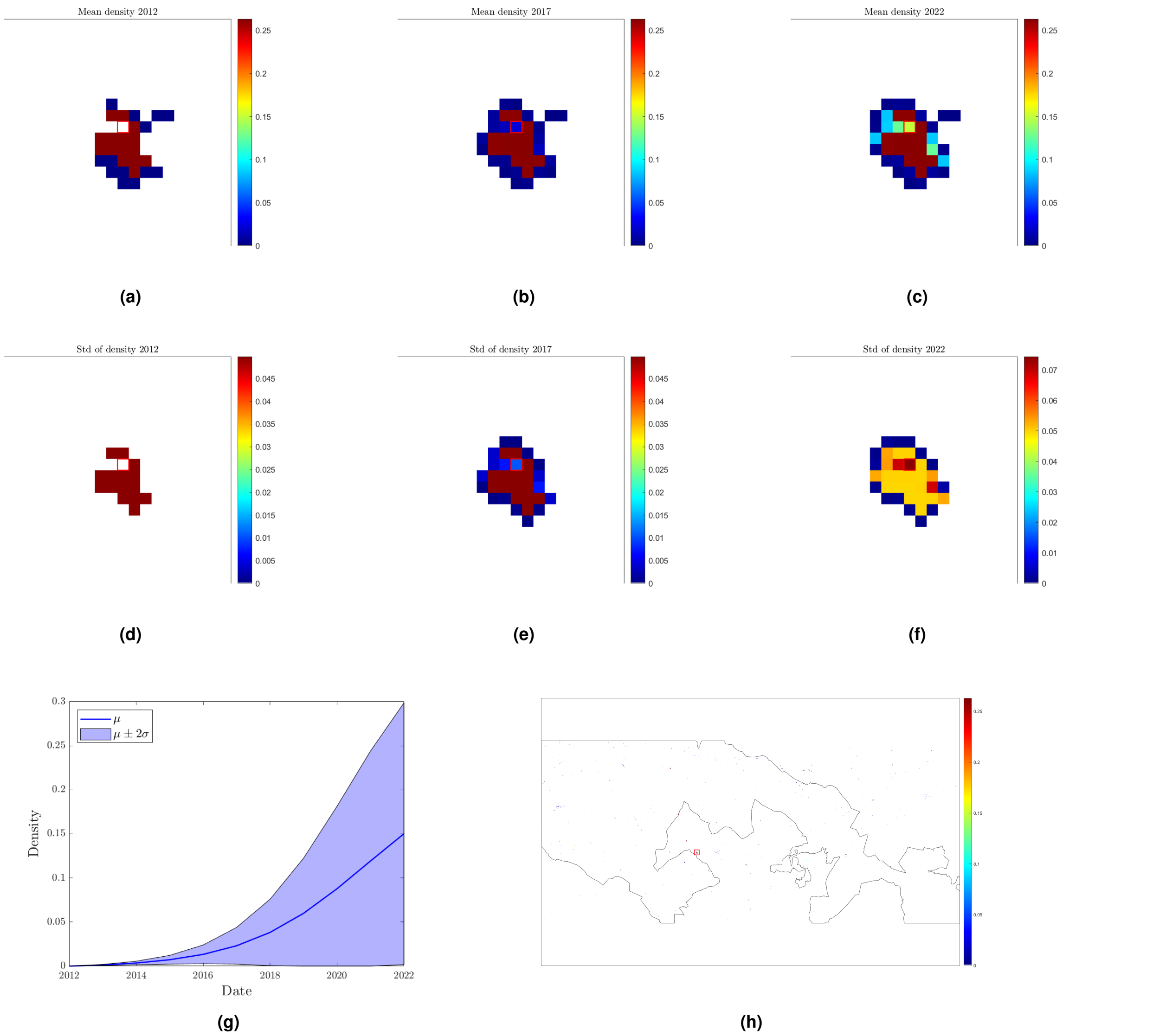

Figure 3. Mean (a)-(c) and standard deviation (d)-(f) of density with time for one cell, highlighted in red (h).

- D: diffusive coefficient. The expert knowledge (LIFE12 BIO/IT/000213) suggests an advancement of *A. altissima* front wave of 2 m per year i.e. an average spread rate equal to $c = 0.002$ km/year. We estimate the diffusive coefficient as[2] $D = \dfrac{c^2}{4r} = 5.2083 \cdot 10^{-7}$ km^2/year.

- k: carrying capacity. According with the expert knowledge (LIFE12 BIO/IT/000213), in a pixel of 4 m^2 (2 m is the remote sensing image very high resolution) can be found at the most two adult plants; here we refer as adult a plant with a trunk of 10 cm of diameter. Consequently, a carrying capacity of $5 \cdot 10^5$ plants per km^2 is considered, i.e. $k = 5 \cdot 10^5$ km^{-2}.

- τ: average time for eradicating a single plant. An item of control (a team of 4 person) is estimated to be able to eradicate approximately 1000 plant a day. Hence, the average time for eradicating a single plant is 1/1000 of day i.e. $\tau = 1/(1000 \cdot 365) = 2.7397 \cdot 10^{-6}$ year.

- μ: harvesting rate per population density unit, due to control. Note that the control action contemplates not only the eradication process but all the action related to reach and cleaning the zone. A team is supposed to clean an area of $2 \cdot 10^{-4}$ km^2 at the carrying capacity in one day. We may estimate μ by imposing that after one day, the

number of plant $U(1/365)$ is less than one:

$$U(1/365) \approx U(0) - \frac{1}{365}\frac{\mu U(0)}{1+\tau\mu U(0)} << 1$$

Here $U(0) = \int_{\Omega,|\Omega|=2\cdot 10^{-4}} k\,d\mathbf{x} = k\cdot 2\cdot 10^{-4} = 100$ plants. Hence $\mu > 365\cdot 99/100/(1+365\cdot 99\cdot\tau) = 328.7989$. Here we set $\mu = 10^4$ year^{-1} so that, when expressed with respect to the densities, $\mu = 10^4\cdot 2\cdot 10^{-4} = 2$ km^{2}year^{-1}.

- $\rho(\mathbf{x})$: habitat suitability. It is an dimensionless coefficient.

Objective function parameters selection

The cost function can be written as

$$\begin{aligned}\mathfrak{J}(E) &= \alpha\int_0^T e^{-\delta t}\left(\int_\Omega E^2(\mathbf{x},t)\,d\mathbf{x}\right)dt + \beta\int_0^T e^{-\delta t}\left(\int_\Omega \frac{E^3(\mathbf{x},t)}{B^3}d\mathbf{x}\right)dt \\ &+ \gamma\int_0^T e^{-\delta t}U(t)\,dt + \theta\, e^{-\delta T}U(T),\end{aligned}$$

where $U(t) = \int_\Omega u(\mathbf{x},t)\,d\mathbf{x}$ represents the number of plant in Ω at time t.

Here α and γ represent the (non discounted) annual cost in in a unitary area (1 km^2) related to an individual team and caused by an individual invasive respectively, ν is a weight for the final population, B is the budget constraint and $\delta\in(0,1)$ is the discount factor. Take into account that in our model the effort powers are amplifying factors which do not alter their dimensionality (for example E^2 is to intended as the density E times a dimensionless coefficient equal to the numerical value of E)

- δ: the discount factor. The discount factor is determined by the European Central Bank for adjusting the effect of the inflation. We approximate the discount factor δ with the mean value of the inflation in the period 2014-2017 according to the HICP index (Harmonized Inflation Consumer Prices), i.e. $\delta = 0.0044$ year^{-1}.

- α: the annual cost related to an individual team in the unitary area. The daily cost of a single team (comprehensive of the herbicide costs) to clean an area (at its carrying capacity) of 200 mt$^2 = 2\cdot 10^{-4}$ km^2 is about €420. Hence

$$\begin{aligned}&\alpha\int_0^{1/365} e^{-\delta t}\left(\int_{\Omega,|\Omega|=2\cdot 10^{-4}}\left(\frac{1}{2\cdot 10^{-4}}\right)^2 d\mathbf{x}\right)dt = 420\\ &\alpha\int_0^{1/365} e^{-\delta t}\,dt = 840\cdot 10^{-4}\\ &\alpha = \frac{840\cdot 10^{-4}\,\delta}{1-e^{-\delta/365}}\end{aligned}$$

It follows that $\alpha = 30.6602$ € day^{-1}, i.e. $\alpha = 11190.973$ € year^{-1}.

We may evaluate the annual density cost of control of *A. altissima* tree per km^{-2}, taking into account that 5000 teams a day, in one day are needed, hence $5000/365 = 13.6986$ teams a day for one year. Hence

$$\alpha\int_0^1 e^{-\delta t}\int_{\Omega,|\Omega|=1}(13.6986)^2\,dx\,dt \approx 2.1\cdot 10^6.$$

- β: the penalty term. We assume $\beta = 11190.973$ € year^{-1} km^{-2}.

- B the budget constraint. We suppose to penalize the choice of more than 2 teams per the total of infested area. From results of the Life Alta Murgia Project about the mapping of *A. altissima* in the Alta Murgia National Park, it results that the total of infested area is about 2258099 m^2, i.e. 2.25801 km^2, hence $B = 2/2.25801 = 0.88574$ km^{-2}.

- γ and θ: costs caused by damages caused by an individual invasive (annual and at final time T). Of the total cost of 12.5 billion euros' in the EU related to management of invasive species as estimated in 2008, a cost of about 9.6 billion euros' each year (i.e. about 76.8 percent) are related to damages caused by invasive alien species in terms of health care and animal health, crop-yield losses, damage to infrastructure, protected species and so forth[3].

  If we assume that the same ratios between the control and damages costs in UE holds in the Alta Murgia National Park with respect to the *A. altissima* costs, we can roughly estimate damages costs as follows. Life Alta Murgia Project costs related to eradication of *A. altissima* indicates a control cost of about $2.3\cdot 10^6$ euros per km^2. If we assume that it corresponds to the 23.2 percent of the total cost, we evaluate as $7.6138\cdot 10^6$ euros per km^2 the costs due to the damages of *A. altissima* w.r.t. the total costs for its managing.

  Let us consider the term

$$\gamma\int_0^T e^{-\delta t}U(t)dt=\gamma\int_0^T\left(e^{-\delta t}U(t)-e^{-\delta T}U(T)\right)dt+\gamma T e^{-\delta T}U(T).$$

  We recognize in $\omega T e^{-\delta T}U(T)$, the contribute of this term to the annual costs related to the final presence $U(T)$. In our model this contribute has to be added to the term $\theta e^{-\delta T}U(T)$ i.e. $(\gamma T+\theta)e^{-\delta T}U(T)$ identifies the total cost related to a non null presence of invasive at the final time. In order to estimates these two parameters by means of an unique datum, we may suppose that θ is a multiple of γT, say $\theta=m\gamma T$. In so doing, we can evaluate γ form the relation

$$\frac{\gamma}{\delta}(1-e^{-\delta})k+m\gamma e^{-\delta}k=7.6138\cdot 10^6,$$

  i.e.

$$\gamma=\frac{7.6138\cdot 10^6\,\delta}{k[1+(m\delta-1)e^{-\delta}]}.$$

  In our model we assume $m=1$ so that $\gamma=7.639$ € year^{-1} and $\theta=7.639$ €.

Value	Parameter	Ecological meaning	Dimension	Ref.
D	$5.2083\cdot 10^{-7}$	diffusion coefficient	km^2 year^{-1}	(*),([2])
r	1.92	growth rate	year^{-1}	([1])
k	$5.0\,10^5$	carrying capacity	km^{-2}	(*)
μ	2	harvesting rate	km^2 year^{-1}	(*)
τ	$2.7397\cdot 10^{-6}$	average time for eradicating a single plant	year	(*)
δ	$4.4000\,10^{-3}$	discount factor	year^{-1}	(**)
α	11190.973	annual cost for a single team	€ year^{-1}	(*)
B	0.88574	maximum effort density	km^{-2}	(*)
β	11190.973	annual penalty cost in a unitary area	€ year^{-1} km^{-2}	assumed
γ	7.639	annual cost for a single invasive plant	€ year^{-1}	(*),([3])
θ	7.639	cost for a single invasive plant	€	(*)

Table 4. Parameters values and related dimensions. (*) The parameters have been here estimated based on the available data and expertise of the project team involved in Life Alta Murgia Project. (**) Mean value of the inflation in the period 2014-2017 according to the HICP index (European Central Bank). For comprehensive information regarding the estimated parameters, please refer to the accompanying text where a detailed description is provided

Model implementation

In this supplementary informations, we give more details about the model (1)-(2) of the paper, and describe the numerical method implemented in the R® code COINS.R (COntrol of INvasive Species), available from https://github.com/CnrIacBaGit/COINSvlabrepo.

First of all we normalize the model and derive the state-adjoint system to be discretized as well as the optimality condition.

The normalized version of the model, in terms of the variable $n = u/k$, finds the optimal effort allocation, E($\mathbf{x}$, t), which minimizes

$$\begin{aligned}\mathfrak{J}(E) &= \int_0^T e^{-\delta t}\left(\int_\Omega E^2(\mathbf{x},t)d\mathbf{x}\right)dt + c\int_0^T e^{-\delta t}\left(\int_\Omega \frac{E^3(\mathbf{x},t)}{B^3}d\mathbf{x}\right)dt \\ &+ \omega\int_0^T e^{-\delta t}\int_\Omega n(\mathbf{x},t)d\mathbf{x}\,dt + \nu e^{-\delta T}\int_\Omega n(\mathbf{x},T)d\mathbf{x}.\end{aligned}$$

subject to the system dynamics

$$\frac{\partial n}{\partial t}(\mathbf{x},t) - D\Delta n(\mathbf{x},t) = r\,n(\mathbf{x},t)\big(\rho(\mathbf{x}) - n(\mathbf{x},t)\big) - \frac{\mu n(\mathbf{x},t)E(\mathbf{x},t)}{1+h\mu n(\mathbf{x},t)}$$

where the previous variable are linked to the novel ones by means of the following relations: $h = \tau k$, $\omega = \dfrac{\gamma k}{\alpha}$, $\nu = \dfrac{\theta k}{\alpha}$, $c = \beta/\alpha$. The sensitivity equation and the adjoint problem yield the following boundary value system[4]

$$\frac{\partial n}{\partial t} = D\Delta n + r\,n\big(\rho - n\big) - \frac{\mu n E}{1+h\mu n} \tag{S.2}$$

$$\frac{\partial \lambda}{\partial t} = -D\Delta\lambda + (\delta - r\rho)\lambda + 2rn\lambda + \frac{\mu\lambda E}{(1+h\mu n)^2} - \omega \tag{S.3}$$

with $n(\mathbf{x},0) = n_0(\mathbf{x})$, $\lambda(\mathbf{x},T) = \nu e^{\delta T}$ and zero-flux boundary conditions. Moreover, $E(\mathbf{x},t) = \min\{\varphi_\chi(n(\mathbf{x},t),\lambda(\mathbf{x},t)),B\}$, on $\Omega\times[0,T]$ where $\chi = 3c/B^3$and the corresponding optimal control function $\varphi_\chi$is given by

$$\varphi_\chi(s,z) = \frac{1}{\chi}\left(\sqrt{1+\frac{\chi\mu s z}{1+h\mu s}} - 1\right). \tag{S.4}$$

We normalize the cost function w.r.t. α, and with abuse of notations we set $\mathfrak{J} = \mathfrak{J}/\alpha$. $N(t) = \int_\Omega n(\mathbf{x},t)d\mathbf{x}$ and the novel parameters are defined as follows: $\omega = \dfrac{\gamma k}{\alpha} = 341.3018$ km^{-2}, $\nu = \dfrac{\theta k}{\alpha} = 341.3018$ year km^{-2}, $c = \beta/\alpha = 1$ km^{-2}, $h = \tau k = 1.37$ km^{-2} year. . In Table 5 we resume all the parameters related to the normalized optimality system (S.2)-(S.3)-(S.4).

Parameter	Value	Dimension
D	$5.2083\cdot10^{-7}$	km^2 year^{-1}
r	1.92	year^{-1}
μ	2	km^2 year^{-1}
h	1.37	year km^{-2}
δ	$4.4\cdot10^{-3}$	year^{-1}
B	0.88574	km^{-2}
c	1	km^{-2}
ω	341.3018	km^{-2}
ν	341.3018	year km^{-2}

Table 5. Normalized parameters used in simulation and related dimensions.

Numerical approximation

The optimality system is solved using the forward-backward sweep method[5–7].

1. Perform the spatial discretization of Equations (S.2) and (S.3) using finite differences in two dimensions in a rectangular grid embedded in the work area. Write a system of M ordinary differential equations, one for each grid point, in vectorial form

$$\mathbf{N}'(t) = L\mathbf{N}(t) + \mathbf{F}(\mathbf{N}(t),\mathbf{V}(t)), \qquad 0\le t\le T, \tag{S.5}$$

$$\mathbf{V}'(t) = -L\mathbf{V}(t) + \delta\mathbf{V}(t) + \mathbf{G}(\mathbf{N}(t),\mathbf{V}(t)), \qquad 0\le t\le T, \tag{S.6}$$

$$\mathbf{N}(0) = \mathbf{N}^{(0)}, \quad \mathbf{V}(T) = \mathbf{V}^{(T)}.$$

where $\mathbf{N}^{(0)}$ contains the initial density values at each grid point, $\mathbf{V}^{(T)}$ is the vector of constant entries $V_i^{(T)} = \nu e^{\delta T}$ for $i=1,\ldots M$ and L is a $M \times M$ matrix representing the discretized Laplacian in two dimensions by the five-points central formula and implementing zero Neuman boundary conditions as in[8]. Moreover,

$$\mathbf{F}_i(\mathbf{N}(t),\mathbf{V}(t)) = r\,N_i\big(\rho_i - N_i\big) - \frac{\mu N_i E_i}{1+h\mu N_i} \qquad i=1,\ldots,M,$$

$$\mathbf{G}_i(\mathbf{N}(t),\mathbf{V}(t)) = -r\,\rho_i\,V_i + 2\,r\,N_i\,V_i + \frac{\mu V_i E_i}{(1+h\mu N_i)^2} - \omega, \qquad i=1,\ldots,M,$$

and the control vector $\mathbf{E}$ has entry

$$E_i = \min\left\{ \frac{1}{\chi}\left(\sqrt{1+\frac{\chi\,\mu N_i\,V_i}{1+h\,\mu\,N_i}} - 1\right), B \right\} \tag{S.7}$$

for each grid point inside the protected area and $E_i = 0$ outside.

2. Use a vector with entries $V_i = \nu e^{\delta T}$ as guess function $\mathbf{V}(t)$ for all $0 \le t \le T$.
3. Solve Eq. (S.5) forward in time, using the initial condition $\mathbf{N}(0)=\mathbf{N}^{(0)}$ and the guess function $\mathbf{V}(t)$.
4. Solve Eq. (S.6) backwards in time using the final condition $\mathbf{V}(T)=\mathbf{V}^{(T)}$ and the solution of Eq. (S.5).
5. Update the control using Eq. (S.7).
6. Repeat steps 3-5 until $\mathbf{N}$, $\mathbf{V}$ and $\mathbf{E}$ converge in the chosen vectorial norm.

For performing points 3 and 4 above, we consider the composition of the first order Lawson symplectic scheme[9] denoted as $\Phi_{\Delta t}$, for solving the local reaction dynamics, with Implicit Euler scheme $\Psi_{\Delta t}$, for the only diffusive terms on a temporal grid of amplitude Δt. The resulting scheme $\Psi_{\Delta t} \circ \Phi_{\Delta t}$ in the forward-backward formulation, solves $\mathbf{N}$ forward in time according to the following $n = T/\Delta t$ steps:

$$\frac{\mathbf{N}_{l_1} - \mathbf{N}_l}{\Delta t} = \mathbf{F}(\mathbf{N}_l, \mathbf{V}_{l_1}), \qquad \mathbf{N}_{l+1} = (I - \Delta t\, L)^{-1}\mathbf{N}_{l_1} \quad l = 0,\ldots,n-1.$$

Then it solves for $\mathbf{V}$, backward in time, by means of exponential Lawson symplectic counterpart:

$$\mathbf{V}_{l_1} = (I - \Delta t\, L)^{-1}\mathbf{V}_{l+1}, \qquad \mathbf{V}_l = e^{-\delta\Delta t}\big(\mathbf{V}_{l_1} - \Delta t\,\mathbf{G}(\mathbf{U}_l, \mathbf{V}_{l_1})\big), \quad l = n-1, n-2,\ldots,0.$$

References

1. Bullock, J. M. *et al.* Modelling spread of british wind-dispersed plants under future wind speeds in a changing climate. *J. Ecol.* **100**, 104–115 (2012).

2. Murray, J. Temperature-dependent sex determination (tsd): Crocodilian survivorship. *Math. Biol. I. An Introd.* 119–145 (2002).

3. Scalera, R., Genovesi, P., Essl, F. & Rabitsch, W. *The impacts of invasive alien species in Europe* (European Environment Agency EEA Technical report No 16/2012, 2012).

4. Baker, C. M., Diele, F., Marangi, C., Martiradonna, A. & Ragni, S. Optimal spatiotemporal effort allocation for invasive species removal incorporating a removal handling time and budget. *Nat. Resour. Model.* **31**, e12190 (2018).

5. Lenhart, S. & Workman, J. T. *Optimal control applied to biological models* (CRC press, 2007).

6. Martiradonna, A., Diele, F. & Marangi, C. Optimal control of invasive species with budget constraint: qualitative analysis and numerical approximation. *Curr. Trends Dyn. Syst. Biol. Nat. Sci.* 147–164 (2020).

7. Ragni, S. A constructive method for parabolic equations with opposite orientations arising in optimal control. *J. Math. Analysis Appl.* **512**, 126092 (2022).

8. Garvie, M. Finite-difference schemes for reaction–diffusion equations modeling predator–prey interactions in matlab. *Bull. mathematical biology* **69**, 931–56, DOI: 10.1007/s11538-006-9062-3 (2007).

9. Diele, F., Marangi, C. & Ragni, S. Exponential Lawson integration for nearly hamiltonian systems arising in optimal control. *Math. Comput. Simul.* **81**, 1057–1067, DOI: https://doi.org/10.1016/j.matcom.2010.10.010 (2011). Important aspects on structural dynamical systems and their numerical computation.
